# Doublet Metalens with Simultaneous Chromatic and Monochromatic Correction in the Mid-Infrared

**DOI:** 10.3390/s22166175

**Published:** 2022-08-18

**Authors:** Yi Zhou, Fengyuan Gan, Ruxue Wang, Dun Lan, Xiangshuo Shang, Wei Li

**Affiliations:** 1 State Key Laboratory of Functional Materials for Informatics, Shanghai Institute of Microsystem and Information Technology, Chinese Academy of Sciences, Shanghai 200050, China; 2 University of Chinese Academy of Sciences, Beijing 100049, China

**Keywords:** metasurface, doublet metalens, mid-infrared, achromatic

## Abstract

Metalenses provide a powerful paradigm for mid-infrared (MIR) imaging and detection while keeping the optical system compact. However, the design of MIR metalenses simultaneously correcting chromatic aberration and off-axis monochromatic aberration remains challenging. Here, we propose an MIR doublet metalens composed of a silicon aperture metalens and a silicon focusing metalens separated by a fused silica substrate. By performing ray-tracing optimization and particle-swarm optimization, we optimized the required phase profiles as well as the sizes and spatial distributions of silicon nanopillars of the doublet metalens. Simulation results showed that the MIR doublet metalens simultaneously achieved chromatic and off-axis monochromatic aberration reduction, realizing a continuous 400 nm bandwidth and 20° field-of-view (FOV). Thanks to its planar configuration, this metalens is suitable for integration with CMOS image sensor to achieve MIR imaging and detection, which has potential application in troubleshooting and intelligent inspection of power grids. This work may facilitate the practical application of metalens-integrated micro/nanosensors in intelligent energy.

## 1. Introduction

The mid-infrared (MIR) region usually refers to wavelengths from 3 to 5 μm, which covers one of the atmospheric transmission windows, rotational–vibrational spectrum of most molecules, and spectrum of high-temperature objects. Therefore, it is widely used in night vision, infrared remote sensing, free-space communications, molecular fingerprint detection, and troubleshooting of power grids [1]. However, the traditional MIR optical lenses are bulky and heavy, thus become the limiting factor for the miniaturization of optical systems in practical applications.

Compared with the traditional counterparts, metasurfaces provide a novel but powerful paradigm for optical wave-front control while keeping the optical system compact. Metasurfaces are artificially designed functional devices with subwavelength micro/nanostructures arranged in a specific way on a two-dimensional plane [2], and are able to engineer the amplitude, phase, polarization, and spin/orbital angular momentum of incident waves by purposely adjusting the size, orientation, and spatial distribution of subwavelength structures. Various functional devices utilizing metasurfaces have been reported, such as metalenses [3,4,5,6,7,8,9,10,11,12,13,14,15,16], beam steering [17,18,19,20], holograms [21,22,23,24,25], polarizers [26,27,28,29,30,31,32], and vortex-beam generators [33,34,35,36,37,38,39].

Though MIR metalenses [40,41,42,43,44,45,46] have shown great potential in compact optical systems, it is still a challenge for metalenses to be further utilized in practical applications, due to the existence of chromatic aberration and off-axis monochromatic aberration, which significantly decrease the focusing and imaging performance of optical systems.

Efforts have been made in the designs of achromatic metalenses in the MIR region [42,43,44,45,46]. In 2019, Zhou et al. reported an achromatic MIR metalens from 3.7 to 4.5 μm using the Pancharatnam–Berry phase and the propagation phase to control the wave front of light and eliminate the chromatic aberration, respectively [42]. In 2021, Ou et al. reported broadband achromatic MIR metalenses from 3.5 to 5 µm by achieving on-demand phase-dispersion control range through choosing much more guided modes supported by taller nanopillars [43]. In the same year, Li et al. reported an achromatic bifocal MIR metalens with polarization sensitivity over a continuous wave band from 3.9 to 4.6 µm [44]. In 2022, Xiong and Sha et al. reported polarization-independent broadband achromatic MIR metalenses that covered continuous bands in 3–5 μm by using the phase compensation of the specially designed cross unit cells [45,46]. However, the above reports only corrected the chromatic aberration of MIR metalenses. The correction of off-axis monochromatic aberration of MIR metalenses has not been reported yet. To date, wide field of view (FOV) metalenses are mostly in the visible and near-infrared (NIR) region [5,6,47,48,49]. The design of MIR metalenses simultaneously correcting chromatic aberration and off-axis monochromatic aberration remains challenging.

A doublet metalens composed of two metalenses located at the front side and back side of the substrate and can correct the aberration through the phase compensation of two metalenses may provide a solution for simultaneous chromatic and monochromatic correction. In some reports, these metalenses at the front and back were named aperture metalens (or correcting metalens) and focusing metalens, respectively [5,6]. Thanks to its sandwich structure, which fully utilizes both sides of the substrate, a doublet metalens is as thin as a singlet metalens. Doublet metalenses have been reported in the visible and NIR region, correcting either the off-axis monochromatic aberration or the chromatic aberration, thus realizing wide-FOV imaging [5,6], broadband achromatic focusing [50], and multiwavelength wide-FOV focusing [51,52].

In this letter, we propose and numerically verify an MIR doublet metalens simultaneously realizing chromatic and off-axis monochromatic aberration reduction. The proposed doublet metalens is composed of a silicon aperture metalens and a silicon focusing metalens separated by a fused silica substrate, working as a concave lens and convex lens, respectively. By performing ray-tracing optimization and multiobjective optimization based on a particle-swarm optimization algorithm sequentially, we optimized the required phase profiles of the doublet metalens, as well as the sizes and spatial distributions of silicon nanopillars of the doublet metalens. Simulation results showed that the optimized doublet metalens simultaneously achieved chromatic and monochromatic aberration reduction, realizing achromatic focusing over a continuous 400 nm bandwidth from 3.1 to 3.5 μm and 20° FOV, thus verifying the effectiveness of the proposed optimization method. Thanks to its planar configuration, the metalens is suitable for integration with a CMOS image sensor to achieve MIR imaging and detection, which is useful in monitoring and analyzing the operating status of equipment. This work may facilitate the practical application of metalens-integrated micro/nanosensors in intelligent energy, especially in troubleshooting and intelligent inspection of power grids.

## 2. Design and Optimization of the MIR Doublet Metalens

**Figure 1:**
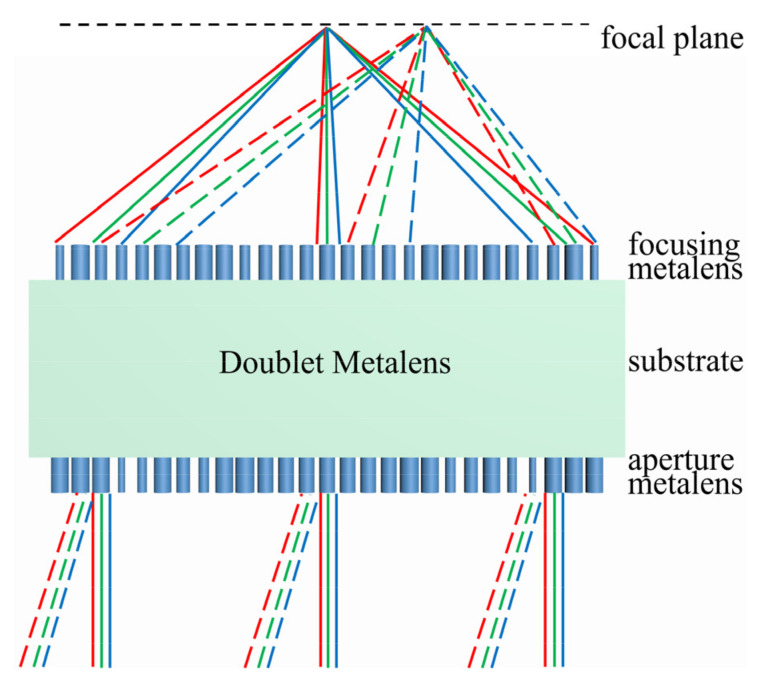
Schematic of the doublet metalens, which simultaneously achieves chromatic and off-axis monochromatic correction.

Figure 1 schematically shows the ray diagram of the doublet metalens realizing achromatic focusing at normal incidence and oblique incidence, which simultaneously achieves chromatic and off-axis monochromatic correction. Incident light propagates through the aperture metalens first, then transmits through the substrate, and finally is focused by the focusing metalens. The broadband incident light with same incident angle is focused on the same spot at the focal plane. The broadband incident light with different incident angles is focused on other spots along the horizontal direction on the same focal plane. Each metalens is composed of thousands of cylindrical silicon nanopillars with the same lattice period and height but different diameters arranged on a tetragonal lattice.

### 2.1. Correction of Off-Axis Monochromatic Aberration

The phase profiles of the aperture metalens and focusing metalens are as follows:

(1) $$\varphi_{A}=\sum_{i=1}^{5}a_{i}{\left(\frac{r}{R}\right)}^{2i},$$

(2) $$\varphi_{F}=\sum_{i=1}^{5}b_{i}{\left(\frac{r}{R}\right)}^{2i},$$

where $\varphi_{A}$ represents the phase profile of the aperture metalens, $\varphi_{F}$ represents the phase profile of the focusing metalens, a_i_ and b_i_ are coefficients of the above polynomials, which are optimized by ray-tracing optimization, $r=\sqrt{x^{2}+y^{2}}$ is the radial coordinate of each nanopost at each metalens, *x* and *y* are position coordinates of each nanopost with respect to the center of each metalens, and *R* is the radius of the doublet metalens [5,6]. The number of coefficients of the above polynomials can be chosen from 3 to 8. Considering the tradeoff between focusing performance and optimization time, the number is set to 5.

**Figure 2:**
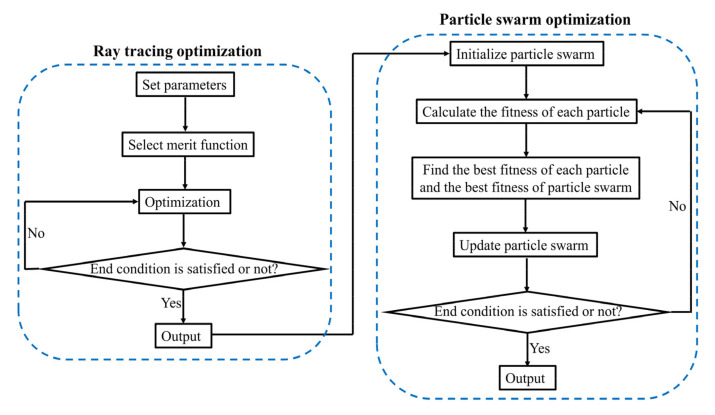
Flowchart of optimization procedures, including ray-tracing optimization and particle-swarm optimization.

**Figure 3:**
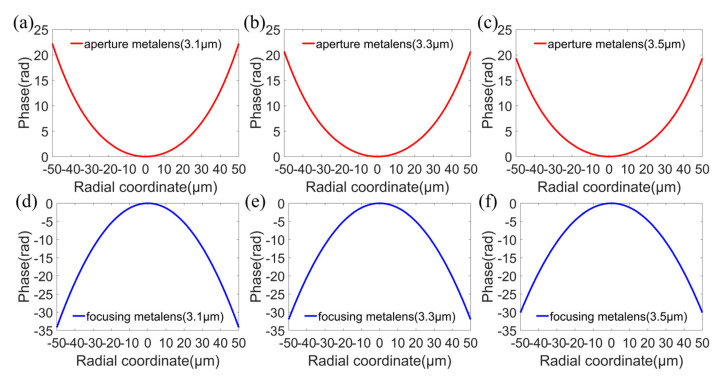
Phase profiles of the aperture metalens (**a**–**c**) and focusing metalens (**d**–**f**) at wavelengths 3.1 μm (lower limit of operating band), 3.3 μm (center wavelength), and 3.5 μm (upper limit of operating band), respectively.

We designed a MIR doublet metalens with a diameter of 100 μm and a focal length of 120 μm, giving NA of 0.38 or f-number of 1.2, with an operating band from 3.1 to 3.5 μm. Here, the substrate is fused silica and the thickness of the substrate is 70 μm. The flowchart of optimization procedures is shown in Figure 2, including ray-tracing optimization and particle-swarm optimization. In the ray-tracing optimization procedure, the initial values of *a_i_* and *b_i_* were all set to zero, for *i* = 1, 2, 3, 4, 5. The target of the ray-tracing optimization is to minimize the focal spots of incident rays with incident angles of 0°, 2.5°, 5°, 7.5°, and 10° at the fixed plane of Z = 120 μm (i.e., the focal plane). The optimizations were carried out at wavelengths of 3.1 μm, 3.2 μm, 3.3 μm, 3.4 μm, and 3.5 μm, respectively. After optimization, the optimized coefficients a_i_ and b_i_ were obtained, and are listed in Table 1 and Table 2, respectively. Phase profiles of the aperture metalens and focusing metalens according to Equations (1) and (2) at wavelengths 3.1 μm (lower limit of operating band), 3.3 μm (center wavelength), and 3.5 μm (upper limit of operating band) are shown in Figure 3a–f, respectively. The phase profiles of the aperture metalens indicate that the aperture metalens works as a concave lens, which diverges incident rays. On the contrary, the phase profiles of focusing metalens indicate that the focusing metalens works as a convex lens, converging rays to the focal plane. The aperture metalens and focusing metalens work together to correct the monochromatic aberrations induced by oblique incidences, thus realizing diffraction-limited focusing on the same focal plane with different incident angles from 0° to 10°, corresponding to 20° FOV. In general, FOV is double the largest incident angle considering the symmetry of metalens [5,6].

### 2.2. Correction of Chromatic Aberration

From Table 1 and Table 2, we know that the phase profiles vary with different wavelengths and cannot be simultaneously satisfied by the same doublet metalens, thus resulting in chromatic aberrations when broadband incident light propagates through the doublet metalens. To be more specific, if we design spatial distribution of silicon nanopillars with various sizes according to the phase profiles of the doublet metalens at one particular wavelength, it cannot satisfy the phase profiles at other wavelengths. Therefore, broadband incident light propagating through the doublet metalens has different foci along the longitudinal direction at different wavelengths, which means chromatic dispersion. In order to minimize chromatic aberrations and thus realize diffraction-limited achromatic focusing, the phase profiles at each wavelength should be engineered to tailor chromatic dispersion.

The required phase profiles of the aperture metalens and focusing metalens to achieve achromatic diffraction-limited focusing for incident light are as follows:

(3) $$\varphi_{A-require}(\lambda ,r)=\varphi_{A}(\lambda ,r){+C}_{A}(\lambda ),$$

(4) $$\varphi_{F-require}(\lambda ,r)=\varphi_{F}(\lambda ,r){+C}_{F}(\lambda ),$$

where *λ* is wavelength, $\varphi_{A-require}(\lambda ,r)$ represents the required phase profile of the aperture metalens at each wavelength, $\varphi_{F-require}(\lambda ,r)$ represents the required phase profile of the focusing metalens at each wavelength, $\varphi_{A}(\lambda ,r)$ represents the obtained phase profile of the aperture metalens at each wavelength based on Equation (1) after ray-tracing optimization, $\varphi_{F}(\lambda ,r)$ represents the obtained phase profile of the focusing metalens at each wavelength based on Equation (2) after ray-tracing optimization, and $C_{A}(\lambda )$ and $C_{F}(\lambda )$ are phase corrections of the aperture metalens and focusing metalens, respectively, which are radial coordinate-independent but wavelength-dependent. The role of $C_{A}(\lambda )$ and $C_{F}(\lambda )$ is to significantly reduce the difficulty of finding an adequate spatial distribution of silicon nanopillars with various sizes that simultaneously satisfies the required phase profiles at all wavelengths in the target operating band [7]. The phase corrections $C_{A}(\lambda )$ and $C_{F}(\lambda )$ are optimized by multiobjective optimization based on a particle-swarm optimization algorithm, which we explain later.

**Figure 4:**
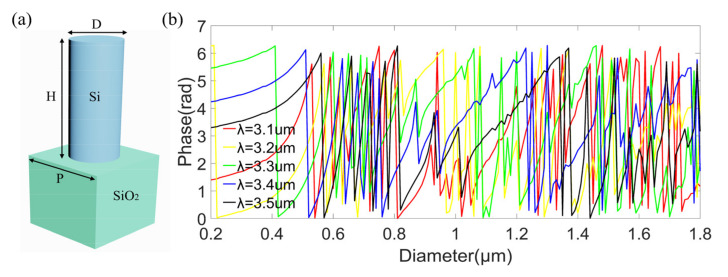
(**a**) Schematic of the unit cell, composed of a silicon nanopillar on fused silica substrate. (**b**) The phase shifts as functions of the nanopillar diameters at wavelengths of 3.1 μm (red line), 3.2 μm (yellow line), 3.3 μm (green line), 3.4 μm (blue line), and 3.5 μm (black line).

Before optimizing the above mentioned phase corrections, we build the database of unit cells of the metalens. Here, the unit cell of the metalens is a silicon nanopillar on a fused silica substrate, shown in Figure 4a, with the size of unit cell (i.e., the lattice period) set as 1.8 μm and the height of the silicon nanopillar set as 8 μm. The target operating band is set from 3.1 to 3.5 μm and discretized into five equally spaced wavelengths. The phase shifts were calculated by the finite-difference time-domain (FDTD) method using the commercial simulation software EastWave, with the nanopillar diameters swept from 0.2 to 1.8 μm in steps of 10 nm. The incident source in the simulation is an x-polarized plane wave. The simulation boundary is periodic in x-direction and y-direction, and perfectly matched layer (PML) in z-direction. The calculated phase shifts (folded between 0 and 2π) as functions of the nanopillar diameters at wavelengths of 3.1 μm (red line), 3.2 μm (yellow line), 3.3 μm (green line), 3.4 μm (blue line), and 3.5 μm (black line) are shown in Figure 4b, clearly showing that both multiple 2π phase coverage and anomalous dispersions are achieved at these wavelengths, which are crucial to the realization of broadband achromatic metalens [7]. Here, multiple 2π phase coverage provides more choices to find adequate silicon nanopillars in the database to meet the phase requirements at different wavelengths. That is to say, silicon nanopillars with several different diameters provide the same phase shift at one wavelength (e.g., 3.1 μm), while silicon nanopillars with these diameters provide different phase shifts at another wavelength (e.g., 3.5 μm). Therefore, we can choose one of these diameters to meet both the required phases at wavelengths of 3.1 μm and 3.5 μm. Furthermore, we can choose one diameter to meet all the required phases at wavelengths from 3.1 to 3.5 μm (in steps of 0.1 μm), as long as the database is rich enough. Besides, the abovementioned anomalous dispersions provide another degree of freedom to design the metalens. Here, anomalous dispersions mean that unfolded phase shifts do not always decrease or increase monotonically with nanopillar diameters, while phase shifts at some diameters experience a contrary variation tendency to most others.

After building the above database of unit cells of the metalens, the phase corrections $C_{A}(\lambda )$ and $C_{F}(\lambda )$ were optimized by multiobjective optimization based on particle-swarm optimization. In the optimization procedure, the initial values of $C_{A}(\lambda )$ and $C_{F}(\lambda )$ were all set to zero, for *λ* = 3.1 μm, 3.2 μm, 3.3 μm, 3.4 μm, and 3.5 μm. Then, $C_{A}(\lambda )$ and $C_{F}(\lambda )$ were optimized separately. At each cycle of the optimization, the sizes and spatial distributions of silicon nanopillars at the aperture metalens or focusing metalens were designed according to both the database and the required phase profiles at wavelengths from 3.1 to 3.5 μm (in steps of 0.1 μm) based on Equation (3) or Equation (4). To be specific, at each cycle of the optimization, the design of the sizes and spatial distributions of silicon nanopillars and the calculation of phase difference is as follows. At each position $r_{m}$ of the aperture metalens or focusing metalens, at each wavelength $\lambda_{n}$, the absolute difference between the phase in the database and the required phase based on Equation (3) or Equation (4) is calculated as:

(5) $$\Delta \varphi_{m,n,k}=|\varphi_{required}(r_{m},\lambda_{n})-\varphi_{database-k}(r_{m},\lambda_{n})|,$$

where subscript *m* represents different positions of the designed aperture metalens or focusing metalens, subscript *n* represents different wavelengths in the operating band, and subscript *k* represents different nanopillars in the database. The phase differences at all wavelengths in the operating band are summed as:

(6) $$\Delta \varphi_{m,k}=\sum_{n}|\varphi_{required}(r_{m},\lambda_{n})-\varphi_{database-k}(r_{m},\lambda_{n})|,$$

**Figure 5:**
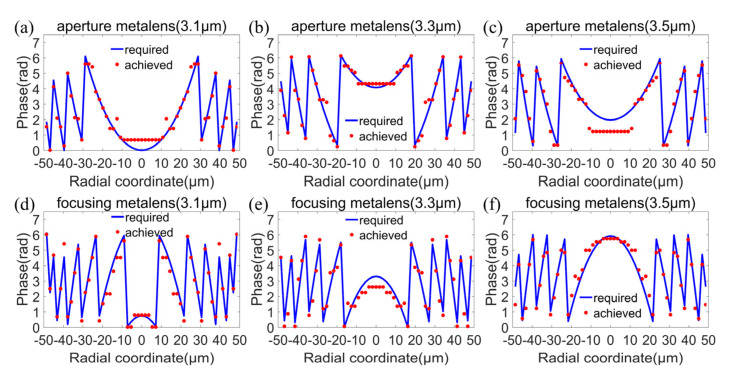
Comparisons of the required phase profiles (blue lines) and the phase profiles induced by the designed aperture metalens and focusing metalens (red dots) at wavelengths 3.1 μm (**a**,**d**), 3.3 μm (**b**,**e**), and 3.5 μm (**c**,**f**), respectively.

At each position, the minimum of $\Delta \varphi_{m,k}$ in the whole database is found, which represents the best optimization between all wavelengths. The corresponding nanopillar in the database is designed at this position. The phase induced by the designed nanopillar at each position and at each wavelength is $\varphi_{metalens}(r_{m},\lambda_{n})$. Then, the total phase difference (referred to as $\Delta \varphi_{total}$ in Equation (7)) between the required phase profiles based on Equation (3) or Equation (4) (referred to as $\varphi_{required}$ in Equation (7)) and the phase profiles induced by the designed aperture metalens or focusing metalens (referred to as $\varphi_{metalens}$ in Equation (7)) at all positions and all wavelengths were calculated at each cycle of the optimization. The total phase difference is calculated as follows:

(7) $$\Delta \varphi_{total}=\sum_{m}\sum_{n}|\varphi_{required}(r_{m},\lambda_{n})-\varphi_{metalens}(r_{m},\lambda_{n})|,$$

where subscript *m* represents different positions of the designed aperture metalens or focusing metalens and subscript *n* represents different wavelengths in the operating band. The target of the optimization is to minimize the total phase difference. By comparing the total phase difference at each cycle with the minimum value of the total phase difference in previous cycles, we updated the values of $C_{A}(\lambda )$ or $C_{F}(\lambda )$ based on the rules of the particle-swarm optimization algorithm and repeated the above procedures. After multiple optimization cycles, the optimal values for phase corrections $C_{A}(\lambda )$ or $C_{F}(\lambda )$ were obtained while the total phase difference was minimized. The phase corrections of the aperture metalens and focusing metalens are listed in Table 3 and Table 4, respectively. The comparisons of the required phase profiles (blue lines) and the phase profiles induced by the designed aperture metalens and focusing metalens (red dots) as functions of radial coordinates at wavelengths 3.1 μm, 3.3 μm, and 3.5 μm are shown in Figure 5a–f, respectively. As is clear from Figure 5, both the designed aperture metalens and focusing metalens satisfy the required phase profiles well. The average differences (abbreviated to ave. dif. in Table 3 and Table 4) between the required phase profiles and the phase profiles induced by the designed aperture metalens and focusing metalens at each wavelength are calculated and listed in Table 3 and Table 4, respectively. Setting 2πrad as the unit, the average differences between the required phase profiles and the phase profiles induced by the designed aperture metalens and focusing metalens are merely 0.0527 and 0.0588 at 3.1 μm, 0.0352 and 0.0472 at 3.2 μm, 0.0519 and 0.0721 at 3.3 μm, 0.0589 and 0.0684 at 3.4 μm, and 0.0643 and 0.0733 at 3.5 μm. According to the well-known Rayleigh criterion, the optical system is perfect when the maximum wave-front aberration is less than a quarter of the wavelength. That is to say, the imaging quality of the optical system is not significantly different from that of the ideal optical system. Here, the average difference is equivalent to the wave-front aberration in the Rayleigh criterion. Such small average differences (all much less than a quarter of wavelength) verify that the designed doublet metalens satisfy the required phase profiles well, and thus can realize simultaneous reduction of both chromatic and off-axis monochromatic aberrations.

## 3. Results

**Figure 6:**
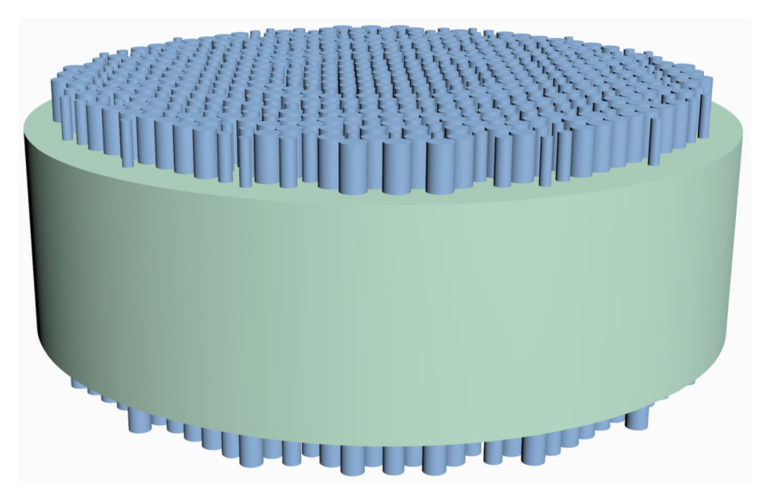
Schematic of three-dimensional structure of the doublet metalens, composed of a silicon aperture metalens and a silicon focusing metalens separated by a fused silica substrate.

**Figure 7:**
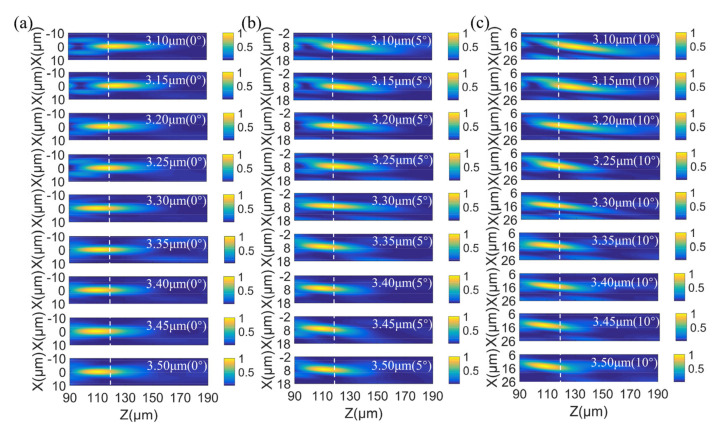
Focusing performance of the doublet metalens. Simulated intensity profiles in the xz-plane at wavelengths from 3.1 to 3.5 μm, with incident angles of 0° (**a**), 5° (**b**), and 10° (**c**), respectively.

The schematic of the three-dimensional structure of the doublet metalens is shown in Figure 6, composed of a silicon aperture metalens and a silicon focusing metalens separated by a fused silica substrate. We analyzed the focusing performance of the designed metalens by means of FDTD method using the abovementioned commercial simulation software EastWave. The incident source in the simulation is an x-polarized plane wave. The simulation boundary is PML in x-direction, y-direction, and z-direction. The light-intensity profiles of the incident light propagating through the doublet metalens at wavelengths from 3.1 to 3.5 μm (in steps of 50 nm) with incident angles of 0°, 5°, and 10° were simulated. The simulated normalized intensity profiles near the foci in the xz-plane at each wavelength with incident angles of 0°, 5°, and 10° are shown in Figure 7a–c, respectively, where the Z-axis is the optical axis and the X-axis both the radial direction of the metalens and the projection direction of oblique incidents on the metalens. As is clear from Figure 7, at the fixed position of z = 120 μm (dashed line in Figure 7), which is the designed focal length, foci are formed over the whole operating band, 3.1–3.5 μm, and over all incident angles, 0°–10°. The variations in focal lengths over the operating band 3.1 to 3.5 μm are small, with standard deviations of 5.12 μm, 5.90 μm, and 7.18 μm with incident angle, of 0°, 5°, and 10°, respectively. The reason for the increasing variations in focal length with the increase in incident light angle is that the off-axis monochromatic aberration increases with the increase of incident angle, thus the difficulty in simultaneously correcting the chromatic and off-axis monochromatic aberrations increases, resulting in the increasing variations of focal lengths over the operating band. According to the simulation results, the effectiveness of the above optimization method is verified. The optimized doublet metalens has simultaneously achieved chromatic and off-axis monochromatic aberration reduction, realizing a continuous 400 nm bandwidth and 20° FOV.

**Figure 8:**
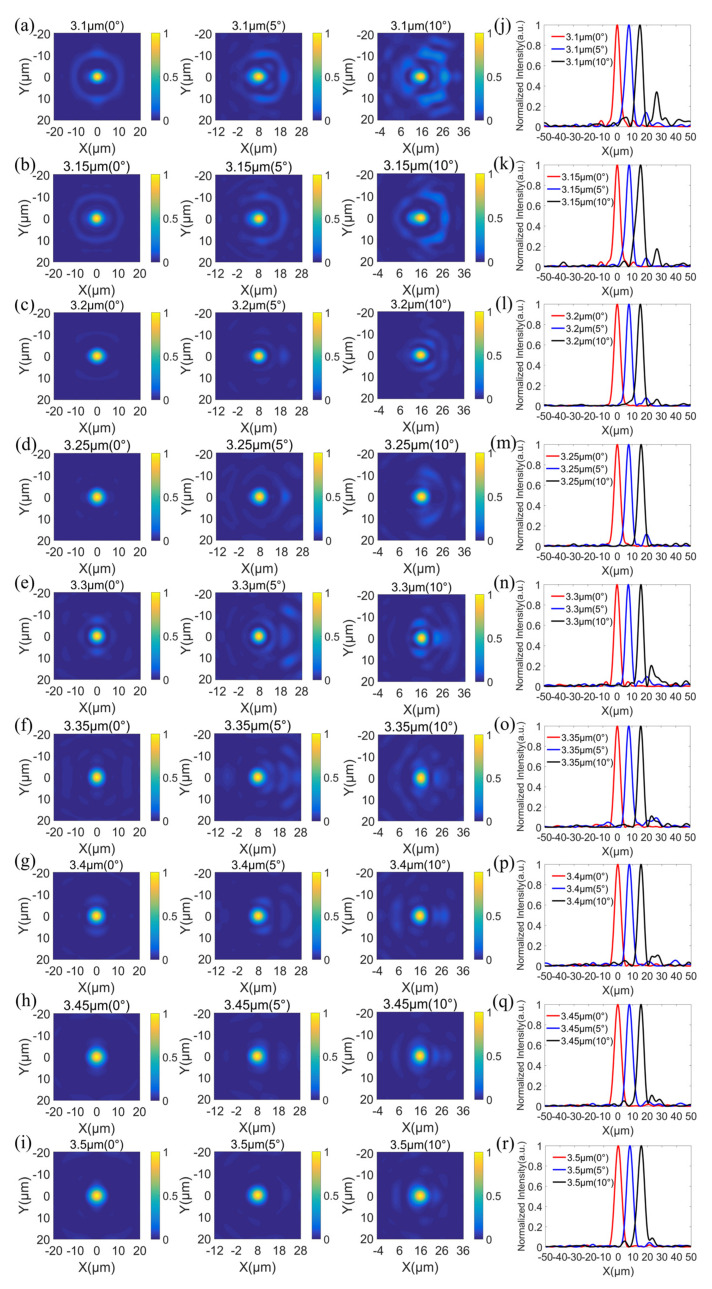
Focusing performance of the doublet metalens. (**a**–**i**) Simulated intensity profiles in the xy-plane at a fixed position of z = 120 μm, i.e., the predesigned focal plane, at wavelengths from 3.1 to 3.5 μm with incident angles of 0°, 5°, and 10°, respectively. (**j**–**r**) Simulated intensity distributions along the X direction of the focal planes at wavelengths from 3.1 to 3.5 μm with incident angles of 0° (red), 5° (blue), and 10° (black), respectively.

In order to further verify the focusing performance of the designed doublet metalens, the normalized intensity profiles in the xy-plane at the fixed position of z = 120 μm (i.e., the predesigned focal plane) with incident angles of 0°, 5°, and 10° at wavelengths from 3.1 to 3.5 μm (in steps of 50 nm) were simulated and are shown in Figure 8a–i, respectively. The strong focal spots with relatively weak sidelobes over the whole operating band 3.1–3.5 μm and over all incident angles 0°–10° are clearly shown in these figures. The reason for the weak sidelobes is that the normalized intensity profiles shown in Figure 8 with incident angles of 0°, 5°, and 10° at wavelengths from 3.1 to 3.5 μm are all at the same fixed plane of z = 120 μm, which is the predesigned focal plane, but it slightly deviates the exact focal planes. At some wavelengths and at some incident angles, the focal lengths slightly deviate the predesigned focal length, as mentioned above in the variations of focal lengths, thus the xy-plane at the fixed position of z = 120 μm slightly deviates the exact focal planes, resulting in weak sidelobes. The horizontal cuts of the focal plane at wavelengths from 3.1 to 3.5 μm (in steps of 50 nm) with incident angles of 0° (red line), 5° (blue line) and 10° (black line) are shown in Figure 8j–r, respectively. As is clear from Figure 8, the focal spots of different wavelengths in the operating band are located at the same focal plane and nearly same horizontal position in the focal plane, while the focal spots of different incident angles are located at the same focal plane but shift along X direction (i.e., the projection direction of oblique incidences on the metalens). Based on the intensity distributions from Figure 8j–r, the shifts of focal spots along X direction and the full widths at half-maximum (FWHMs) of focal spots at each wavelength and each incident angle are calculated and listed in Table 5 and Table 6, respectively. According to Table 5, the shifts of focal spots along the X direction with incident angle of 5° over the operating band 3.1 to 3.5 μm are nearly the same, with an average shift of 7.81 μm and a standard variation of merely 0.13 μm. Similarly, the variation in the shifts of focal spots along the X direction with incident angle of 10° over the operating band 3.1–3.5 μm is quite small, with an average shift of 15.87 μm and a standard variation of merely 0.23 μm. As per Table 6, the FWHMs of focal spots are close to λ/2NA, revealing diffraction-limited focusing performance of the doublet metalens. Therefore, the simultaneous reduction in chromatic and off-axis monochromatic aberration by the designed doublet metalens is further verified.

To practically obtain the proposed doublet metalens, the fabrication technique of doublet metalens should be developed. The practicable fabrication steps are as follows. First, amorphous silicon is deposited on both sides of the fused silica substrate by chemical vapor deposition (CVD). Second, location markers are fabricated by lithography at both sides of the wafer. Third, patterns of the aperture metalens are developed on the basis of the location markers on one side of the wafer by electron-beam lithography (EBL) and a hard mask is developed on the pattern. Fourth, the pattern of the focusing metalens developed on the basis of the location markers on the other side of the wafer by electron-beam lithography and hard mask is developed on the pattern, while protecting the aperture metalens pattern. Fifth, etching of aperture metalens and focusing metalens are done by inductively coupled plasma (ICP) in turn. After these steps, the doublet metalens is fabricated and can be used in practical applications.

## 4. Conclusions and Discussion

We have proposed and numerically verified an MIR doublet metalens composed of a silicon aperture metalens and a silicon focusing metalens separated by a fused silica substrate, working together to reduce off-axis monochromatic aberration and chromatic aberration. Each metalens is composed of thousands of cylindrical silicon nanopillars. By performing ray-tracing optimization and particle-swarm optimization sequentially, we optimized the required phase profiles of the doublet metalens, as well as the sizes and spatial distributions of the silicon nanopillars. Simulation results show that the optimized MIR doublet metalens has simultaneously achieved chromatic and off-axis monochromatic aberration reduction, realizing a continuous 400 nm bandwidth from 3.1 to 3.5 μm and 20° FOV, thus verifying the effectiveness of the proposed optimization method. Compared with traditional bulky MIR lenses, the proposed MIR doublet metalens provides a novel but powerful paradigm for chromatic and monochromatic correction, while keeping the optical system compact. The doublet metalens proposed in this work is as compact as a singlet metalens, with a size comparable to the previous MIR singlet metalenses, which merely achieved achromatic focusing [42,43,44,45,46]. Thanks to its planar configuration, this metalens is suitable for integration with a CMOS image sensor to achieve micro/nanosensors to monitor and analyze the operating status of equipment. This work may facilitate the practical application of metalens-integrated micro/nanosensors in intelligent energy, especially in troubleshooting and intelligent inspection of power grids.

We believe that the bandwidth and FOV can be further increased by several methods. Here, the unit cell of the metalens is simply a pillar with the shape of a cross-section as a circle. If we utilize unit cells with multiple cross-section shapes, such as annular pillar, concentric pillar, square pillar, square annular pillar, and cross-shaped pillar, the database including phase and phase dispersion will be enlarged, thus supporting larger bandwidth [8]. Furthermore, if free-form unit cells generated by generative adversarial network (GAN) are utilized, the database and the bandwidth will be enlarged further. The FOV of the doublet metalens can be further increased by increasing the diameter ratio between the focusing metalens and aperture metalens, which is limited by our computational resources in this work. If the diameter ratio between the focusing metalens and aperture metalens is increased, incident light at larger oblique angles can reach within the range of focusing metalens and be effectively focused by the focusing metalens, and thus can realize a wider FOV up to 50° or 60° by the abovementioned ray-tracing optimization [5,6]. It is worth noting that, given the same optical aperture, which means the diameter of the aperture metalens is kept unchanged, increasing the diameter ratio between the focusing metalens and aperture metalens not only requires more computational resources by squared growth but also raises the difficulty of maintaining the same operating bandwidth. Similarly, the problem of operating bandwidth can be solved by the abovementioned database-enlargement method.

## Figures and Tables

**Table 1:** Phase-profile coefficients *a_i_* of the aperture metalens at wavelengths from 3.1 to 3.5 μm.

Wavelengths (μm)	*a_1_*	*a_2_*	*a_3_*	*a_4_*	*a_5_*
3.1	16.758	4.346	1.194	0.815	−0.880
3.2	16.160	4.360	1.247	0.095	−0.377
3.3	15.844	2.340	4.177	−1.145	−0.549
3.4	14.870	4.874	0.709	−1.203	0.751
3.5	14.832	2.078	4.071	−1.109	−0.543

**Table 2:** Phase-profile coefficients *b_i_* of the focusing metalens at wavelengths from 3.1 to 3.5 μm.

Wavelengths (μm)	*b_1_*	*b_2_*	*b_3_*	*b_4_*	*b_5_*
3.1	−33.489	−1.043	0.443	−0.134	0.018
3.2	−32.418	−0.998	0.422	−0.127	0.017
3.3	−31.347	−0.980	0.418	−0.127	0.017
3.4	−30.394	−0.947	0.404	−0.123	0.017
3.5	−29.476	−0.918	0.392	−0.120	0.017

**Table 3:** Phase corrections and average differences of the aperture metalens at wavelengths from 3.1 to 3.5 μm.

Wavelengths (μm)	3.1	3.2	3.3	3.4	3.5
$C_{A}(\lambda )$ (rad)	0.0105	8.076	4.076	2.765	−4.311
Ave. dif. (2π rad)	0.0527	0.0352	0.0519	0.0589	0.0643

**Table 4:** Phase corrections and average differences of the focusing metalens at wavelengths from 3.1 to 3.5 μm.

Wavelengths (μm)	3.1	3.2	3.3	3.4	3.5
$C_{F}(\lambda )$ (rad)	13.315	1.857	−2.980	4.941	−6.645
Ave. dif. (2π rad)	0.0588	0.0472	0.0721	0.0684	0.0733

**Table 5:** The shifts of focal spots along X direction at the focal plane at wavelengths from 3.1 to 3.5 μm.

Wavelengths (μm)	3.1	3.15	3.2	3.25	3.3	3.35	3.4	3.45	3.5
X (μm) (0°)	0	0	0	0	0	0	0	0	0
X (μm) (5°)	7.85	8.05	7.87	7.69	7.57	7.75	7.78	7.80	7.90
X (μm) (10°)	15.43	15.88	15.67	16.19	16.18	16.02	15.86	15.84	15.72

**Table 6:** The FWHMs of focal spots at wavelengths from 3.1 to 3.5 μm.

Wavelengths (μm)	3.1	3.15	3.2	3.25	3.3	3.35	3.4	3.45	3.5
FWHM (μm) (0°)	4.35	4.56	4.91	4.83	4.60	4.66	5.03	5.41	5.32
FWHM (μm) (5°)	5.33	4.84	4.62	4.83	4.30	4.96	4.88	5.10	5.16
FWHM (μm) (10°)	5.19	5.70	4.77	4.83	4.16	4.51	4.57	4.64	5.48

## Data Availability

The data presented in this study are available on request from the corresponding author.
